# Use of patient-reported outcomes (PRO) data to complement exposure–response analysis in early clinical cancer drug development

**DOI:** 10.1186/s41687-023-00651-2

**Published:** 2023-11-17

**Authors:** Huiming Xia, Brian P. Booth, Yaning Wang, Chunling Fan, Vishal Bhatnagar, Paul Kluetz, Jeanne Fourie Zirkelbach

**Affiliations:** 1 Bristol Myers Squibb, Princeton, USA; 2 Division of Cancer Pharmacology I, Office of Clinical Pharmacology, Office of Translational Sciences, CDER, US Food and Drug Administration, Silver Spring, USA; 3 Greaterna Science and Technology, Shanghai, China; 4 AstraZeneca, Gaithersburg, USA; 5 Oncology Center of Excellence, Office of New Drugs, CDER, US Food and Drug Administration, Silver Spring, USA; 6 Division of Cancer Pharmacology II, Office of Clinical Pharmacology, Office of Translational Sciences, CDER, US Food and Drug Administration, FDA White Oak Campus, 10903 New Hampshire Avenue, Silver Spring, MD 20903 USA

**Keywords:** Patient reported outcomes, Exposure–response analysis, Drug development, Dosage optimization, Adverse events

## Abstract

**Background:**

This proof-of-concept retrospective case study investigated whether patient-reported outcomes (PRO) instruments, designed to capture symptomatic adverse event data, could identity a known exposure–response (ER) relationship for safety characterized in an original FDA analysis of an approved anti-cancer agent. PRO instruments have been designed to uniquely quantify the tolerability aspects of exposure-associated symptomatic adverse events. We explored whether standard ER analyses of clinician-reported safety data for symptomatic adverse events could be complemented by ER analysis using PRO data that capture and quantify the tolerability aspects of these same symptomatic adverse events.

**Methods:**

Exposure-associated adverse event data for diarrhea were analyzed in parallel in 120 patients enrolled in a clinical trial using physician reported Common Terminology Criteria for Adverse Events (CTCAE) and patient-reported symptomatic adverse event data captured by the National Cancer Institute’s (NCI) PRO Common Terminology Criteria for Adverse Events (PRO-CTCAE) instrument. Comparative ER analyses of diarrhea were conducted using the same dataset. Results from the CTCAE and PRO-CTCAE ER analyses were assessed for consistency with the ER relationship for diarrhea established in the original NDA using a 750-patient dataset. The analysis was limited to the 120-patient subset with parallel CTCAE and PRO-CTCAE assessments.

**Results:**

Within the same 120-patient dataset, ER analysis using dense, longitudinal PRO-CTCAE-derived data was sensitive to identify the known ER relationship for diarrhea, whereas the standard CTCAE based ER analysis was not.

**Conclusions:**

ER analysis using PRO assessed symptomatic adverse event data may be a sensitive tool to complement traditional ER analysis. Improved identification of relationships for safety, by including quantification of the tolerability aspect of symptomatic adverse events using PRO instruments, may be useful to improve the sensitivity of exposure response analysis to support early clinical trial dosage optimization strategies, where decision making occurs within limited small patient datasets.

## Background

Since 2010, 27% of new drug approvals by the US Food and Administration (FDA) have been oncology drugs, a proportion that doubled from the previous decade [1]. Advances toward precision medicine with new therapeutic targets that precisely modulate basic biological drivers of disease, and innovative regulatory tools such as Fast Track, Breakthrough Therapy, Priority Review and Accelerated Approval pathways, have significantly contributed to efficient development and rapid approval of oncology products. Also contributing, are novel “seamless” designs aimed at allowing trials to function as registrational trials by incorporating dose escalation and dose expansion cohorts that provide the recommended phase 2 dosage (RP2D) and evidence of efficacy and safety needed to support accelerated approval in many cases. Overall, these innovations in regulatory pathways and clinical trial designs are replacing distinct phase 1, 2 and 3 clinical trials and shortening drug development and review timelines [2–4]. One challenge that remains with seamless designs is that there are less clinical data available within a single first-in human trial to identify dosages that may be optimized and moved forward in larger expansion cohorts or trials designed to support a marketing application.

Current initiatives at the FDA Oncology Center of Excellence include adding patient-centric drug development strategies into clinical trials to complement traditional measures of survival, clinician-reported safety and anti-tumor data. Use of patient reported outcome (PRO) data is one such example. The inclusion of a PRO strategy in a clinical trial can add value by providing scientifically rigorous patient-generated data, leading to a more detailed understanding of symptoms and function that further characterizes clinical benefits and risks. These PRO tools can help investigators assess symptoms and functional impacts of the disease, the treatment, or both [5]. One PRO tool that is increasingly incorporated in clinical trials is the PRO Common Terminology Criteria for Adverse Events (PRO-CTCAE) [6]. The PRO-CTCAE item library was rigorously developed and validated by the National Cancer Institute to assess patient-reported symptomatic adverse events (AEs) in clinical trials, and to complement standard clinician-reported adverse events as assessed by standard CTCAE safety [5, 7].

Based on the expected toxicity profile of the cancer therapies under investigation, a concise set of PRO-CTCAE symptomatic items can be presented to patients at regularly scheduled intervals throughout a trial. This produces a rich set of patient-reported symptom data covering multiple aspects of common symptomatic AEs such as frequency, severity, and interference. Dense, longitudinal data derived from PRO-CTCAE can describe the onset, magnitude of change and trajectory of symptomatic AEs. Used in conjunction with standard clinician-reported CTCAE, the PRO-CTCAE can capture a more robust symptomatic AE data stream without increasing patient sample size and may improve exposure–response (ER) relationships for particular symptomatic AEs. This strategy is consistent with the Oncology Center of Excellence Project Optimus initiative to reform dosage optimization in oncology drug development. Strategies within this initiative include consideration of the patient perspective through inclusion of patient-reported outcomes data to enhance the assessment of tolerability in early phase dosage finding trials. These data should be used in combination with all other relevant nonclinical and clinical data, as well as exposure–response relationships for safety and efficacy to select dosages moved forward in trials designed to support marketing applications [8].

While both CTCAE and PRO-CTCAE are used to assess similar symptoms, they differ in multiple ways (see Table 1). The CTCAE and PRO-CTCAE allow capture of different aspects of symptomatic AEs (such as diarrhea) in clinical trials from clinician and patient perspectives, respectively. This combination approach, introduced prospectively in clinical trials, may capture more information about AEs, and aid in characterizing patient-centric ER relationships that may help to better assess tolerability. Analysis of ER relationships using dense data from PRO-CTCAE holds promise to allow exploration or identification of an optimized tolerable dose or schedule within early development in dose escalation and expansion cohorts. It may do this by identifying exposure–response relationships for safety early, particularly given smaller datasets and rapid timelines seen with contemporary drug development.

**Table 1:**
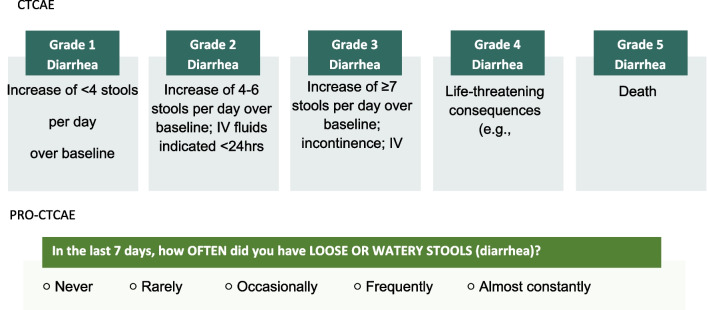
Comparison of CR-diarrhea (CTCAE) and PR-diarrhea (PRO-CTCAE) assessments

Depending on the symptom being assessed, PRO-CTCAE items are used to assess symptomatic AEs along a number of different attributes including frequency, severity, interference, amount, and presence or absence. We conducted a retrospective exploratory analysis to investigate how patient reported (PR) data quantifying diarrhea could complement the ER relationship for safety and tolerability (based on PR data quantifying diarrhea that was previously established during the initial New Drug Application review by FDA).

## Methods

### Study design and patients

FDA previously approved a small molecule anti-cancer agent, based on results from a registrational trial that established its safety and efficacy for patients with a locally advanced metastatic solid tumor. Datasets for assessment of safety, patient reported symptomatic diarrhea and pharmacokinetics (PK) were used in the current retrospective ER analyses.

For the original approval, a total of 750 patients received the study drug following an oral once daily regimen. The dose range investigated in the full dataset ranged from 0.3 to 3 times the recommended dosage. Patients received study drug starting on Day 1 (D1) of Cycle 1 and continued treatment until protocol-defined progression, or until a treatment discontinuation criterion was met. A treatment cycle was defined as 21 days.

Pharmacokinetic sampling was included to assess plasma concentrations of the study drug, with sampling time points on D1 of Cycle 1, Cycle 2 and Cycle 3 (Table 2).

**Table 2:** CR-diarrhea, PR-diarrhea and PK assessment schedules

Week	Pre-screening	Week, treatment period													Follow-up
	0	1	2	3	4–6	7–9	10–12	13–15	16–18	19–21	22–24	25–27	28–30	31–33	33+
Cycle*/Day		C1/D1	C1/D8	C1/D15	C2/D1	C3/D1	C4	C5/D1	C6/D1	C7	C8	C9	C10	C11	
CTCAE	X	X	X	X	X	X	X	X	X	X		X		X	X
PRO-CTCAE		X^a^	X	X	X, X, X	X, X, X	X, X, X	X, X, X	X, X, X	X	X	X	X	X	X
PK Plasma		Pre-dose (± 5 min) 1 h (± 5 min) 2 h (± 10 min) 4 h (± 10 min) 6 h (± 10 min) 8 h (± 10 min)			Pre-dose (± 5 min)	Pre-dose (± 5 min) 1 h (± 5 min) 2 h (± 10 min) 4 h (± 10 min) 6 h (± 10 min) 8h (± 10 min) 10 h (± 10 min) 12 h (± 1 h) 24 h (± 1 h)									

^a^To be completed pre-dose

### CTCAE and PRO-CTCAE assessments

For the current proof of concept retrospective case study, both CTCAE and PRO-CTCAE data were available for a subset of 120 patients within the 750-patient dataset described above. The 120-patient subset included only those patients with parallel CTCAE and PRO-CTCAE data obtained from the registration trail who received study drug at the dosage recommended in the current FDA approved package insert. Furthermore, PRO-CTCAE assessment within the registration trial was only performed in those countries where a linguistically validated version of PRO-CTCAE existed.

Assessments of AEs were performed by clinicians using CTCAE (Version 4.0) from time of informed consent throughout the treatment and safety follow-up periods. The assessment schedule is shown in Table 2. In brief, patients in the trial were assessed for AEs by clinicians on D1 of each 3-week cycle before cycle 7 (up to week 18) and then on D1 of every 2 cycles (every 6 weeks). A sub-set of items from the PRO-CTCAE item bank of symptoms was selected for assessment in the original registrational trial as part of an exploratory objective to assess AEs by PRO for specific CTCAE symptoms. The PRO-CTCAE assessment included a selection of 28 items from the 78 symptomatic AEs within the library. The inclusion of these items was based on the emerging AE profile of the study drug characterized from the earlier clinical studies, preliminary identified study drug exposure response relationships for AEs of interest and patient interviews. The defined recall period for PRO-CTCAE was the past 7 days. Patients completed PRO-CTCAE assessments more frequently than clinician assessments were captured, with PRO-CTCAE assessments occurring weekly up to week 18 and then every three weeks until the end of study (Table 2). The PRO-CTCAE item we analyzed assessed the frequency of diarrhea, asking patients, “In the last 7 days, how often did you have loose or watery stools (diarrhea)?”.

### Clinician reported diarrhea from CTCAE and patient reported diarrhea from PRO-CTCAE

Diarrhea was chosen as the symptomatic AE of interest for the current study, given the safety analysis in the 750-patient dataset for the original approval showing a high incidence of clinician reported diarrhea at any grade from CTCAE, as well as the ER analysis using CTCAE showing increasing incidence of diarrhea at any grade with increasing study drug exposure.

We compared the drug associated diarrhea assessments, using clinician reported (CR)-diarrhea and patient-reported (PR)-diarrhea. CR-diarrhea graded by a clinician incorporates in their assessment frequency, interference with activities of daily living and medical intervention to determine the grade (Table 1). PR-diarrhea response options reported by patients is represented by descriptive frequency (i.e., occasionally) alone (Table 1).

Diarrhea events graded by CTCAE for the study drug were converted to a binary outcome for statistical analysis (presence of diarrhea = 1, and absence = 0). PRO-CTCAE response options for the diarrhea (Loose or watery stools (LWS)) item were coded as follows: never = 0, rarely = 1, occasionally = 2, frequently = 3 and almost constantly = 4, and converted into a binary outcome (Score < 3 = 0, and score ≥ 3 = 1). It is also well documented that patients often report higher severity or incidence of an adverse event using PRO assessment tools, compared to CR-diarrhea scored by CTCAE [8]. A clinically relevant cut-point for diarrhea that would warrant a dose adjustment based on PRO-CTCAE score has not been established in the literature. In this initial exploratory analysis, we considered a PRO-CTCAE frequency score of “frequently” and “almost constantly” (score ≥ 3) as a clinically relevant score that could increase the risk for dose adjustment or clinical intervention by a clinician. As a further analysis PRO-CTCAE response options for diarrhea were also converted into additional binary outcome scores. These cut-points for binary outcomes evaluated as a sub analysis were PRO-CTCAE frequency score cut-points of 4 (“almost constantly”) compared to ≤ 3 (“rarely” to “frequently”) and frequency score cut-points of ≥ 2 (“occasionally” to “almost constantly”) compared to 1 (“rarely”).

### Exposure–response analysis and statistics

CR-diarrhea or PR-diarrhea were included for analysis, regardless of causality, and were evaluated as binary variables. All graphic visualizations and statistical analysis were performed with R (Version 3.6.1). Patients were divided into 4 quartile groups or 2 median groups according to area under the concentration–time curve at steady state (AUCs) for exploring the exposure–response relationships for diarrhea and LWS ≥ 3. The correlation between drug exposure (AUCs) and probability of diarrhea occurrence or achieving a LWS ≥ 3 was estimated by logistic regression. Multivariable logistic regression was conducted to quantify the effect of prior therapy, disease status, baseline chemistry and exposure of study drug by AUCs on CR-diarrhea or PR-diarrhea. Kaplan–Meier analysis of event-free survival and multivariable cox proportional hazards were performed to explore the ER relationship for the time to the first CR-diarrhea or PR-diarrhea. All analyses are ad-hoc and considered to by exploratory and hypothesis generating.

## Results

### Exposure–response for CTCAE diarrhea events

**Fig. 1:**
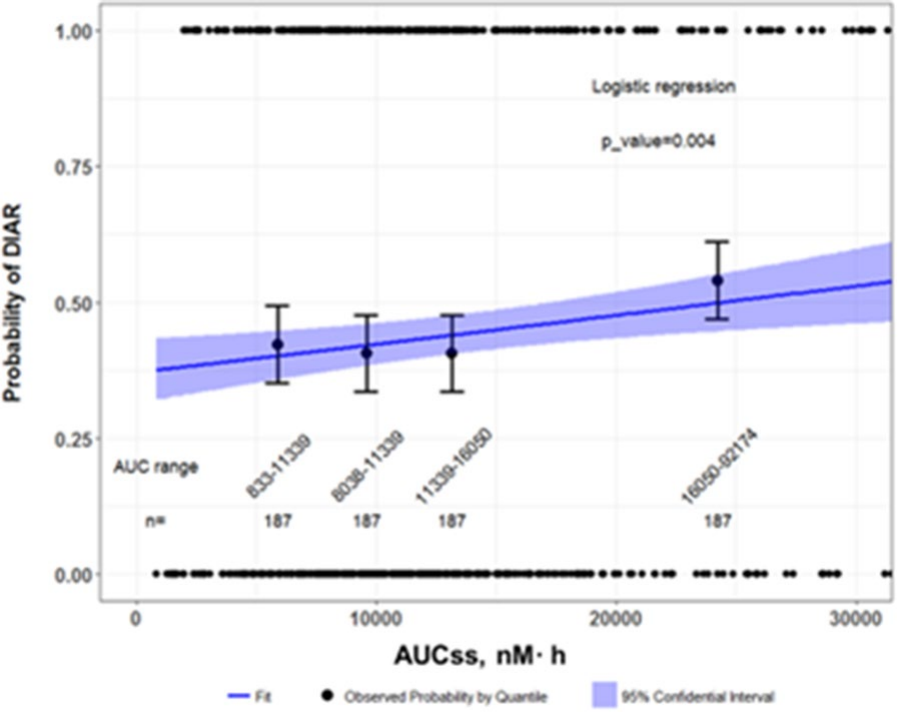
ER analysis of diarrhea with the 750-patient dataset by logistic regression. Observed and model predicated probability of diarrhea occurrence versus AUCs in the complete trial population. CR-diarrhea assessments were available for 750 patients in the ER analysis. Circles indicates the observed diarrhea event (present = 1, absent = 0); vertical bars represent the observed probability of diarrhea in AUC quantile groups; the line and the corresponding band stands for the population probability by logistic regression (logit(P(Y = 1) = − 0.526_ + 2.154e−05*AUCs, *p* = 0.004) and its 95% confidence interval

The original ER analysis for safety to support the original approval of this small molecule anticancer agent showed that diarrhea, as defined by CTCAE at any grade, occurred in 40% of patients within the 750-patient dataset. Based on the original FDA review, the quartile plot of drug exposure (AUCs) and diarrhea showed increasing incidence of diarrhea with increasing study drug exposure (Fig. 1). The logistic regression analysis indicated a relationship between drug exposure at steady state (AUCs) and probability of diarrhea for the overall population (*p* = 0.004, N = 750; Fig. 1). Demographic factors, prior therapies, disease status and baseline laboratory chemistries did not impact diarrhea incidence based on the multivariable logistic regression conducted with these data.

### ER analysis of probability of CR-diarrhea vs probability of PR-diarrhea

**Fig. 2:**
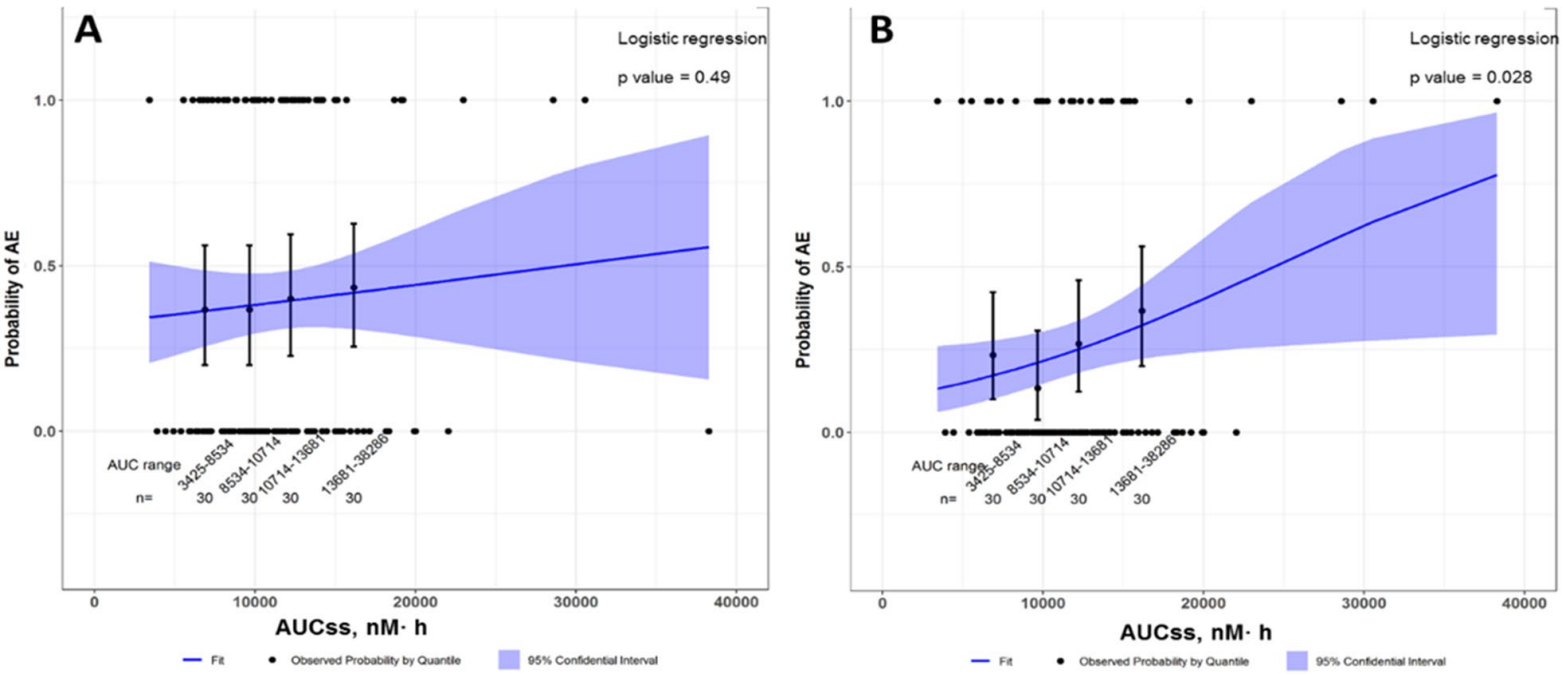
**A** Probability of CR-diarrhea with increasing exposure. **B** Probability of PR-diarrhea with increasing exposure. Observed and model predicated probability of **A** CR-diarrhea or **B** PR-diarrhea versus AUCss of study drug in the subgroup of 120 patients who completed both CTCAE and PRO-CTCAE assessments. Circles indicate the observed event (present = 1, absent = 0); vertical bars represent the observed probability of diarrhea) in AUC quartile groups; the line and the corresponding band stands for the population probability for diarrhea by logistic regression and its 95% confidence interval. The logistic regression model for CR-diarrhea **A** is logit(P(Y = 1) =  − 0 .734_ + 2.499e−05*AUCs, *p* = 0.49; and the model for PR-diarrhea  ≥ 3 is logit(P(Y = 1) = − 0.219_ + 8.992–05*AUCs, *p* = 0.028

The exposure parameter AUCs was selected as the predictor of response for the comparative ER analyses using the 120-patient dataset. The AUCs ranged from 3425 to 38,286 nM∙h following the FDA approved once daily dosing regimen of the study drug. The quartile plot of drug exposure (AUCs) and CR-diarrhea showed a flat relationship of observed diarrhea incidence with exposure (AUCs), while there was a steep increasing incidence of PR-diarrhea with exposure (AUCs) (Fig. 2). By logistic regression, the previously observed ER relationship between drug exposure (AUCs) and the probability of CR-diarrhea was not significant for this subset of the treatment population (*p* = 0.49, N = 120) (Fig. 2A). Conversely, an ER relationship between drug exposure (AUCs) and the probability of PR-diarrhea, was demonstrated, with a nominal *p*-value of 0.028 (Fig. 2B). This relationship between drug exposure and the probability of PR-diarrhea was also demonstrated for the exploratory PRO-CTCAE frequency score cut-points of 4 (“almost constantly”) compared to ≤ 3 (“rarely” to “frequently”) and frequency score cut-points of ≥ 2 (“occasionally” to “almost constantly”) compared to 1 (“rarely”) (data not shown).

### Time to event analysis of first occurrence of CR-diarrhea and PR-diarrhea

**Fig. 3:**
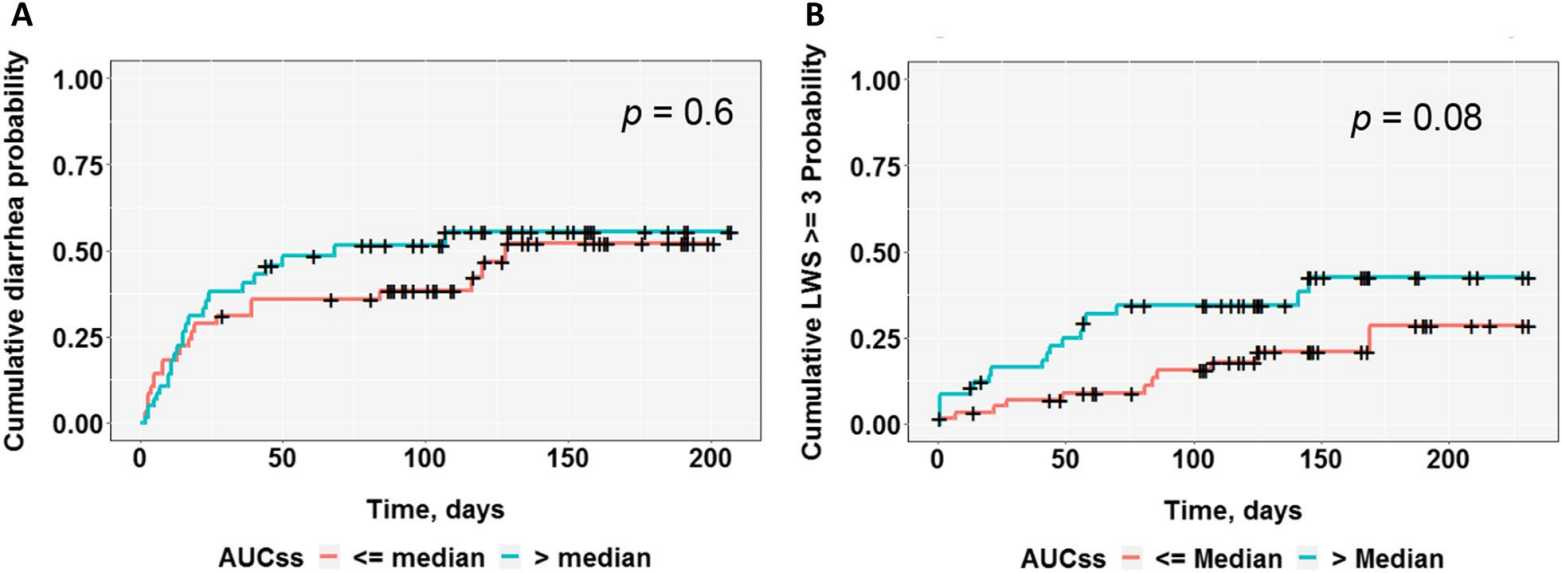
**A** Time to event analysis of ER for CR-diarrhea. **B** Time to event analysis of ER for PR-diarrhea. Cumulative probability of time-to event for **A** CR-diarrhea or **B** PR-diarrhea after the first dose of study drug by Kaplan–Meier analysis in the 120-patient subgroup. Patients were grouped by AUCs median for Kaplan–Meier analysis of time-to-event cumulative probability. The occurrence of any grade CR-diarrhea (CTCAE) or PR-diarrhea (LWS ≥ 3) (frequent or almost constant) was considered an event, and patients with no event were censored and hatch-marked (+) in the plot. Log rank testing did not reveal a robust difference between AUCs median groups from CR-diarrhea (CTCAE) (*p* = 0.6). Log rank testing revealed that the AUCs-upper-median group showed a trend of higher PR-diarrhea (LWS ≥ 3) probability at early stages of treatment, compared to the AUCs-lower-median group (*p* = 0.08)

To explore whether drug exposure (AUCs) affects the time to the first diarrhea event after the first dose of study drug, ER analyses for CR-diarrhea and PR-diarrhea were conducted using a time-to-event approach, where the occurrence of any grade CTCAE diarrhea (CR-diarrhea) or LWS ≥ 3 (PR-diarrhea) was considered an event, and patients with no event were censored. Patients were grouped by median AUCs (patients with AUCs < median AUCs, and patients with AUCs ≥ median AUCs) for a Kaplan–Meier analysis. The cumulative probability plot for CR-diarrhea did not demonstrate a difference between the patients with exposure (AUCs) < the median and exposure (AUCs) ≥ the median (*p* = 0.6; Fig. 3A). The cumulative probability plot for PR-diarrhea showed an early and more consistent separation of LWS ≥ 3 probability over time between the patients with exposure (AUCs) < the median and patients with exposure (AUCs) ≥ the median (*p* = 0.08; Fig. 3B). Multivariable cox proportional hazards regression analyses were conducted to model exposure (AUCs) on any grade CR-diarrhea (CTCAE) or PR-diarrhea (LWS ≥ 3) and it was found that AUCs had a treatment effect on PR-diarrhea but not on CR-diarrhea. The results from the Kaplan–Meier and cox proportional regression analyses suggested that PR-diarrhea may be more sensitive than CR-diarrhea in exploring the ER relationship for this specific previously identified drug exposure associated adverse event of diarrhea.

### Analysis of longitudinal PR-diarrhea

**Fig. 4:**
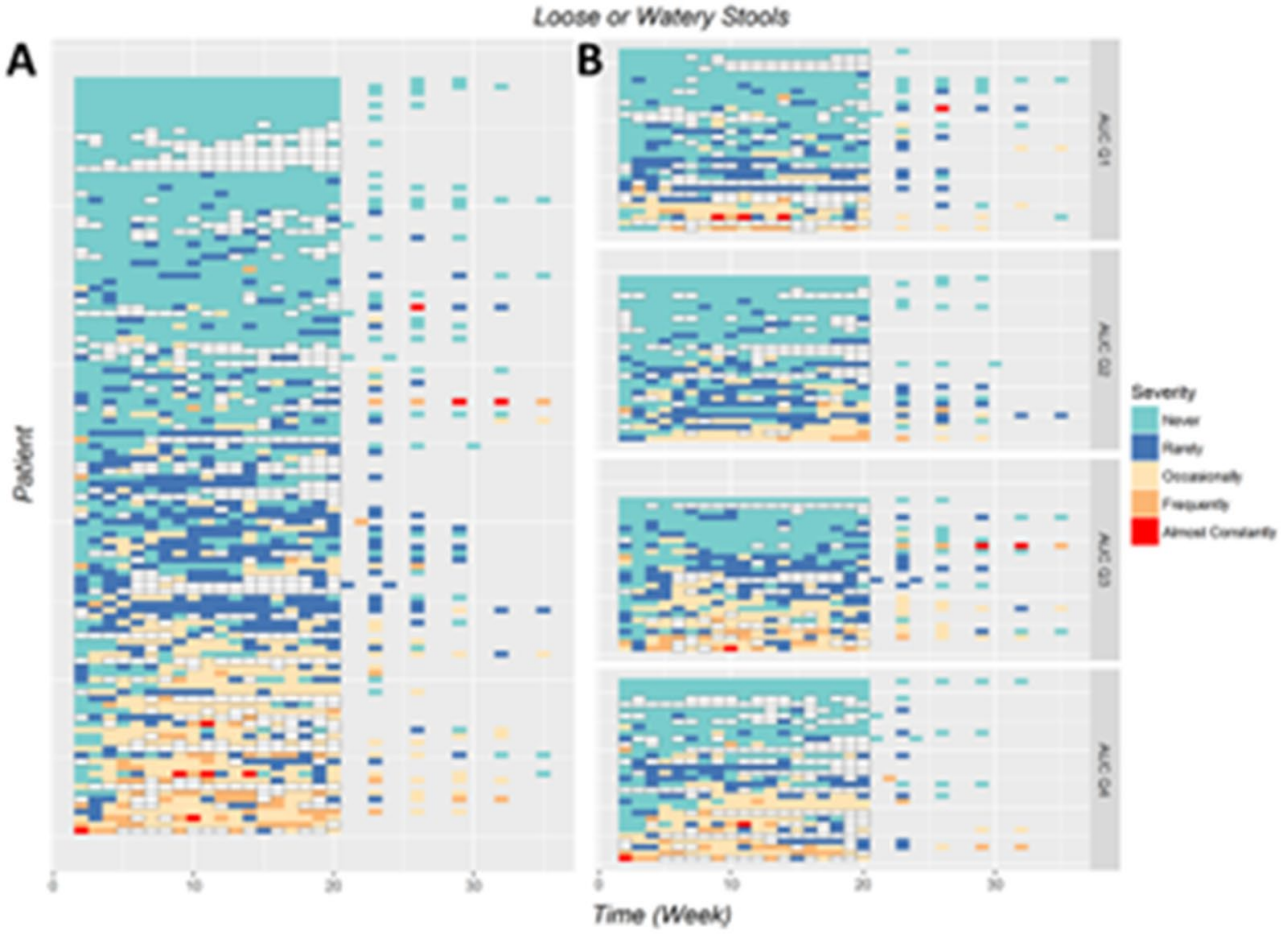
Heatmap of show frequency of PR-diarrhea (LWS) in different AUC quantiles. Longitudinal PR-diarrhea frequency assessed over time versus AUC exposure. The PR-diarrhea frequency over time by heatmap was shown as the whole patient population (**A**) and stratified by drug exposure as AUCs quantile bins (**B**) at individual level. Each line indicates one patient record of PR-diarrhea evaluation. White box indicates a missing observation. Larger areas of orange and red were shown in patients with higher drug exposure (AUCs Q3 andQ4 versus Q1 and Q2)

To explore the ER response of LWS further, a heatmap by exposure quartile (Q1–Q4) was generated to examine the relationship between drug exposure and PRO-CTCAE LWS responses over time (Fig. 4A, B). Results in Fig. 4A display the observed LWS frequency over time for each individual in the 120-patient dataset. Patients were grouped by drug exposure (AUCs) quartiles (Fig. 4B), and a higher frequency of clinically relevant PRO-CTCAE assessed LWS scores ≥ 3 (frequently and almost constantly) was shown in patient groups with highest exposures (AUCs Quartile 3 and Quartile 4). This observation appears consistent with the previously established ER relationship for CTCAE-assessed diarrhea using standard ER analysis in the original 750 patient dataset.

## Discussion

In the current study, ER analyses were repeated using a smaller 120-patient subset of this data for which both CTCAE and PRO-CTCAE were available. Similar to the findings from the ER analysis from the original NDA, the ER analysis using PR-diarrhea assessed by PRO-CTCAE showed a trend of increasing probability of diarrhea (LWS ≥ 3) frequency with increasing steady state exposure (AUCs) (Fig. 2B). However, the ER relationship for CR-diarrhea, assessed by CTCAE, from the same 120 patient dataset did not show a significant relationship (Fig. 2A). The loss of significance in the smaller subset of patients with CTCAE data may be due to the small sample size, compared to the original ER analysis (N = 750). The results suggest that PRO-CTCAE assessing frequency of diarrhea was able to replicate the known ER relationship, and may be more sensitive than CTCAE in identifying this relationship in a smaller sample size using the cutoffs we deployed. These results may suggest possible utility of complementary PRO-CTCAE based ER analyses in the early identification of key adverse events in smaller patient populations enrolled in modern or seamless trial designs, and early application to support dosage selection within key decision windows in dose expansion cohorts. This retrospective analysis serves as a first step to future studies testing PRO-CTCAE-based ER analyses prospectively as a supplementary tool to characterize symptomatic AEs that could affect adherence to cancer treatment.

The findings were also supported by the time to event analysis that showed a trend for a higher cumulative incidence of patient-reported LWS with increased drug exposure that was not clearly observed using CTCAE diarrhea data. These results are consistent with other reports indicating the potential underestimation of the incidence and severity of symptomatic AEs using clinician assessed CTCAE, when compared to patient-reported symptom data [9, 10]. Taken together, Figs. 2B and 3B suggest that the use of PRO-CTCAE in combination with ER using CTCAE may be more sensitive than CTCAE alone in identifying ER relationships for safety.

There are several limitations to our exploratory evaluation of PRO-CTCAE as a tool to complement CTCAE data for safety ER relationships. First, we used a different threshold for our binary cut points for PROCTCAE and CTCAE. For CTCAE we used none versus any grade diarrhea, whereas for PRO-CTCAE we used a higher cut point (frequently or almost constantly versus less than frequently). This was due to the fact there were few patients with grade 3 or higher diarrhea reported by CTCAE. Second, our dataset was relatively small (120 patients) and we know that the ER relationship using CTCAE diarrhea was significant at the full dataset of 750 patients. Nonetheless, the fact that the PRO based analysis did pick up the association with a smaller number of patients suggests that it may be useful in early identification of drug-associated safety signals where only small patient datasets may be available. These preliminary results suggest that ER analysis with added PRO-CTCAE data may be complementary, and hold promise to potentially allow exploration or identification of a more optimized tolerable dose or regimen within dose escalation and expansion cohorts. Prospective studies are needed to further characterize and determine appropriate thresholds to support the potential utility of PRO-CTCAE to inform ER analysis within oncology clinical trial modernization strategies.

PRO-CTCAE data may provide additional advantages over standard CTCAE safety data when included in ER analyses. PRO data are systematically assessed at a certain frequency, generating a larger amount of data on particular symptomatic AEs compared to traditional clinician-based CTCAE evaluations of AE severity. In addition, PRO data may allow for assessment of other aspects of a symptom (e.g., interference), and can also evaluate the effect of toxicity to physical function, ability to work, and other important components of quality of life. In addition, several studies have demonstrated that patients tend to report a higher frequency and severity of symptomatic adverse event data than do clinicians, potentially allowing for improved understanding of tolerability, and early identification of clinically relevant AE signals [6]. Improved tolerability is critical to maximize overall exposure through optimal adherence in an era of increasingly self-administered oral agents. In the current analysis, unique attributes of dense PRO-CTCAE assessments over the treatment period allowed for a visual distribution of LWS frequency over time, which is not available with standard safety tables or ER analysis. Indeed, the FDA Oncology Center of Excellence has recent piloted a website that communicates standard analyses of longitudinal symptomatic adverse events assessed using PRO [11].

We did not see clear patterns of resolution of LWS at the patient level when looking at the heatmap of LWS over time by exposure quartile (Q1–Q4) (Fig. 4). This type of analysis may be useful to include in drug development to assess each individual patient’s trajectory of symptomatic AEs. The timing of symptomatic adverse events and their resolution could be useful in developing patient centric dose adjustment strategies based on the drug specific tolerance patterns observed and effect of the adverse events on physical function and ability to work and perform daily activities.

## Conclusions

PRO-CTCAE is specifically designed for assessment of symptomatic AEs associated with cancer drugs in clinical trials and can be used to generate high-quality data to complement and extend the information provided by standard assessment of symptomatic AEs using CTCAE. However, it has not been determined if PRO-CTCAE can complement CTCAE-based ER analysis for evaluating drug exposure associated symptomatic AEs. The current retrospective analysis supports that ER analyses based on PRO-CTCAE data may provide additional information from a patient’s perspective regarding exposure related AE relationships. If collected early in development, this information can help inform subsequent trial design. Due to advances in treatment, patients with cancer are living longer and receive therapy for longer periods of time. Early detection and characterization of ER relationships for bothersome symptomatic AEs that could affect adherence may aid clinical trial modernization strategies by decreasing uncertainties in selection of optimized dosages within rapid drug development programs. It is acknowledged that within early dosage finding trials, exposure associated symptomatic AEs that may be selected for ER analyses using PRO-CTCAE, may only be identified based on emerging clinical data within the initial dose escalation phase, or within the subsequent activity estimating phase from early trials where longer term safety and tolerability data become available. Therefore, the rationale for specific patient reported symptom selection within early phase trials should be based on a totality of evidence approach and include both mechanistic and early emerging clinical data. Furthermore, patient reported outcomes for implementation in ER analyses based on PRO-CTCAE should focus on specific symptomatic exposure associated AEs (e.g., gastrointestinal symptoms including diarrhea and rash) that occur at high frequency and with increasing drug exposure. Patient reported outcome assessments for ER analyses using PRO-CTCAE may be included within key later portions of early phase clinical trials such as dosage randomization studies in which randomized dosage cohorts are included to evaluate more than one dosage to inform decisions on dosages moved forward in development. For ER analyses based on PRO-CTCAE, it is recommended to select a parsimonious (e.g., 8–12) number of symptom items from instruments such as the European Organization for the Research and Treatment of Cancer Quality of Life Questionnaire (EORTC QLQ-C30) or PRO-CTCAE targeting the expected exposure-associated symptomatic AEs most likely to occur. Open-ended, free-text questions may also be considered to allow for evaluation of patient reported side-effects which are not directly asked with existing PRO items.

There are a growing number of submissions to FDA that include PRO data to support FDA approval. Our initial retrospective analysis suggests that PRO data may be of utility in identifying ER relationships related to safety and tolerability. This work needs to be further explored using larger datasets from different classes of oncology drugs. There is an additional need for development of novel statistical and ER methods for PRO data as well as new data visualization techniques for PRO-CTCAE, which may allow further optimized seamless trial designs and characterization of drug-specific AE fingerprints to aid in dosage selection, and patient-centric dosage adjustment.

## Data Availability

The analyses datasets generated during the current study are not publicly available as the original datasets are part of a New Drug Application submitted to FDA.

## Abbreviations

FDA: US Food and Administration
PRO: Patient-reported outcomes
ER: Exposure response
CTCAE: Common Terminology Criteria for Adverse Events
NCI: National Cancer Institute’s
PRO-CTCAE: PRO Common Terminology Criteria for Adverse Events
RP2D: Recommended phase 2 dosage
AEs: Adverse events
PK: Pharmacokinetics
PR: Patient reported
CR: Clinician reported
LWS: Loose or watery stools
AUCs: Area under the concentration–time curve
EORTC QLQ-C30: European Organization for the Research and Treatment of Cancer Quality of Life Questionnaire
